# Effect of *Bhastrika Pranayama* on neuro- cardiovascular-respiratory function among yoga practitioners

**DOI:** 10.6026/9732063002001549

**Published:** 2024-11-30

**Authors:** Varun Malhotra, Danish Javed, Tanusha Pathak

**Affiliations:** 1 Department of Physiology, AIIMS, Bhopal, India; 2 Department of AYUSH, AIIMS, Bhopal, India

**Keywords:** *Bhastrika Pranayama*, heart rate variability, electroencephalography, sympathetic nervous system, yoga, brain waves, autonomic nervous system physiology

## Abstract

*Bhastrika Pranayama*, a vigorous yoga breathing technique, impacts both the autonomic nervous system and brain function. This prospective interventional study examined the acute effects of *Bhastrika* on heart rate variability (HRV) and electroencephalogram (EEG) patterns in 20 regular yoga practitioners. Significant increases in heart rate and sympathetic activity, as well as alterations in brain wave spectra, were observed during and after the practice indicated by a rise in low-frequency (LF) power and the LF/HF ratio, along with a decrease in high-frequency (HF) power and a substantial decrease in beta, theta, alpha and gamma waves while delta waves increased. The findings suggest *Bhastrika* enhances sympathetic activity and modifies cognitive states. Further research is necessary to understand its long-term benefits and therapeutic potential.

## Background:

The ancient Indian practice of Yoga is widely recognized as a scientific approach to achieving a more complete and meaningful life [1]. In recent years, the benefits of Yoga have been increasingly studied, with research exploring its health advantages for both healthy individuals and those with various medical conditions [2]. Yoga's positive effects are relevant to people of all ages and genders, including young children at formative stages in their lives [3]. "*pranayama*", a term for breathing exercises, is derived from the Sanskrit words "prana" (life force) and "ayama" (control) [4]. It refers to a series of controlled breathing exercises that influence various respiratory parameters, including breathing frequency, inhalation (puraka), retention (kumbhaka), exhalation (rechaka) and body locks (bandhas) [5]. Different types of *pranayama* have distinct effects on the body, depending on the nature and duration of the practice [6]. Some of the common *pranayamas* include Nadishuddhi, Kapalbhati, *Bhastrika* and Bhramari have been shown to impact a wide range of physiological parameters [7]. Evidence suggests that slow, deep breathing positively affects the cardiorespiratory system by lowering blood pressure and heart rate, whereas fast breathing results in a continuous but mild increase in heart rate [8]. A recent study demonstrated that practicing *Bhastrika Pranayama* at a low respiratory rate (RR) reduces heart rate as well as systolic and diastolic blood pressure [9]. This is further evidence that *pranayama* enhances cardiac sympatho-vagal balance and respiratory functions, both of which are crucial for managing psychophysiological stress [10].

Moreover, *pranayama* has been found to positively influence heart rate variability (HRV) by increasing parasympathetic activity, reducing the synthesis of stress hormones and enhancing Gamma-aminobutyric acid (GABA) inhibition from the prefrontal cortex and insula to the amygdala, thereby mitigating the psychological and physical effects of stress [11]. The vagus nerve, which connects the solitary nucleus, thalamus, limbic regions and prefrontal cortex, is believed to be the psychobiological pathway through which *pranayama* exerts its effects [12]. A recent study examined the impact of a thirty-day *Bhastrika Pranayama* training course on a brain network associated with emotion processing, using functional magnetic resonance imaging (fMRI) and self-report questionnaires to assess changes in affect and anxiety [13]. The findings revealed that practicing *Bhastrika Pranayama* for four weeks could reduce anxiety and enhance positive emotions. These changes were correlated with the activity and connectivity of a brain network involved in emotion processing, including the amygdala, anterior cingulate, anterior insula and prefrontal cortex [14]. Therefore, it is of interest to scientifically study and report the acute effects of *Bhastrika Pranayama* on heart rate variability (HRV) and electroencephalogram (EEG) brain spectrum, aiming to observe findings related to the "autonomic-brain axis" or "neurocardiac axis", given the encouraging reports on *Bhastrika*'s impact on HRV.

## Methodology:

## Study setting:

This study was conducted in the Department of Physiology and AYUSH at a tertiary care center in Central India. Prior to commencing the research, ethical approval was obtained from the Institutional Human Ethics Committee, with the reference IHEC No. IM079.

## Study design:

This was a prospective interventional study.

## Study duration:

Two months.

## Sample size:

The sample size for the repeated measures ANOVA analysis was set at 20, assuming an error rate of 0.05, a power of 80% and a medium effect size of 0.3 based on prior studies. This calculation was performed using G*Power software. Twenty healthy, regular yoga practitioners with an average age of 47 years and a normal BMI, who consented to participate, were randomly selected from the Yoga Unit of the AYUSH Department at AIIMS Bhopal.

## Exclusion criteria:

Individuals with compromised pulmonary function, heart conditions, a history of smoking, walking-related dyspnea, pedal edema or high blood pressure were excluded from the study.

## Intervention/procedure:

The *Bhastrika Pranayama* was performed under the supervision of a certified yoga practitioner. Participants were seated comfortably with a straight back and a relaxed posture. They were instructed to sit with their chin parallel to the floor, spine straight, abdomen tucked in and shoulder blades together. The participants were guided through the following steps:

[1] Inhalation: Take a deep breath in through the nose, fully expanding the lungs. The inhalation should be forceful yet not strained, with the abdomen expanding as the breath is taken in.

[2] Exhalation: Exhale forcefully and completely through the nose, contracting the abdominal muscles to push the air out rapidly. A sense of release and a rush of breath leaving the body should be felt.

[3] Rhythm: Continue this forceful inhalation and exhalation in a rhythmic pattern, ensuring the breaths are equal in duration and intensity. Maintain a steady pace without pauses between breaths.

[4] Completion: After completing five minutes of *Bhastrikriya*, participants returned to normal breathing and were asked to sit quietly for about five minutes, observing the effects on their body and mind.

## Precautions:

Participants were advised to avoid excessive practice of this *pranayama* and to perform it only under professional guidance, limiting the duration to five minutes. If any participant reported dizziness, light-headedness, or discomfort during the practice, it was immediately stopped and the participant was instructed to resume normal breathing.

## Measurement of parameters:

Heart rate variability (HRV) was measured using the HRV Brain Tap Dinamika Machine-NEURALCHEK (Braintap INC, New Bern, NC, USA), a cutting-edge digital analyser that evaluates HRV through neurodynamic analysis [15]. This system records an ECG while simultaneously tracking functional state indices in real-time, analyzing a broad range of frequency bands in the human heart rate signal. Both the hardware and software of Dinamika-NEURALCHEK meet the standards for measuring and interpreting cardiac intervalometry indices in clinical practice, as defined by the European Society of Cardiology and the North American Association of Electrophysiology. HRV parameters were assessed after five minutes. The equipment used included two wrist electrodes and a laptop running the mobile HRV NEURALCHEK unit, called "Dinamika". The electrodes were applied to the wrist using jelly or water. Baseline data were collected over five minutes and the parameters were recorded again during and immediately after the five-minute *Bhastrikriya* session while the participant remained in the same position. The brain wave spectrum was also measured during these three intervals.

## Statistical analysis:

Data were tested for normality and found to be parametric, so they are presented as Mean ± SD. Statistical analysis was performed to compare and analyze the heart rate variability, EEG and resting measurements using SPSS for Windows, Version 28 (Statistical Package for the Social Sciences). Repeated measures analysis of variance was conducted. If the p-value for the ANOVA, compared to the F-statistic, was less than 0.05, it was concluded that at least one group was statistically different. Mauchly's test was used to assess the assumption of sphericity and Greenhouse-Geisser or Huynh-Feldt corrections were applied as needed.

## Results:

The study on the acute effects of *Bhastrika Pranayama* reveals significant changes in heart rate variability (HRV) and electroencephalogram (EEG) parameters before, during and after the practice. The heart rate (HR) increased significantly post-*Bhastrika*, indicating heightened cardiovascular activity. Similarly, the SDNN (standard deviation of NN intervals) showed a significant increase during and after *Bhastrika*, reflecting improved autonomic regulation. However, pNN50 and RMSSD, both indicators of parasympathetic activity, decreased significantly post-*Bhastrika*, suggesting a reduction in parasympathetic influence. Interestingly, while the HF (High Frequency) component of HRV, associated with parasympathetic activity, decreased significantly, the VLF (Very Low Frequency) component increased, indicating changes in autonomic regulation. The LF/HF ratio, a marker of sympathovagal balance, increased significantly during *Bhastrika*, suggesting a shift towards sympathetic dominance (Table 1).

In terms of EEG parameters, *Bhastrika Pranayama* led to a significant decrease in theta, alpha, beta and gamma waves, all of which are associated with various states of relaxation, alertness and cognitive processing (Table 2). The most notable decrease was observed in alpha waves post-*Bhastrika*, indicating reduced relaxation or heightened arousal. Conversely, delta waves, typically associated with deep sleep and rest, increased significantly during and after *Bhastrika*, suggesting a shift towards deeper states of relaxation or altered consciousness. *Bhastrika Pranayama* exerts a profound influence on both autonomic nervous system activity and brain function. The practice appears to induce a state of increased sympathetic activity and reduced parasympathetic influence, coupled with significant changes in brain wave patterns that suggest altered states of consciousness. These findings highlight the potential of *Bhastrika Pranayama* as a powerful tool for modulating physiological and neurological states, with implications for both health and wellness.

## Discussion:

*Bhastrika Pranayama* is a yoga breathing technique characterized by forceful and rapid inhalation and exhalation and it is believed to enhance heart rate variability (HRV) [16]. In our study, we observed an increase in both SDNN and total power during *Bhastrika* practice (Table 1), indicating an overall improvement in Heart rate variability (HRV). Higher HRV is generally associated with better cardiovascular health and overall well-being [17]. *Bhastrika Pranayama* has been shown to enhance HRV, suggesting a more flexible and responsive autonomic nervous system [18]. During slow *Bhastrika Pranayama* (fewer than seven breaths per minute), deep and controlled breathing activates the parasympathetic nervous system, which promotes relaxation and rest [19]. However, in the fast and dynamic *Bhastrika Pranayama* practiced in our study (more than seven breaths per minute), there was an increase in sympathetic activity. This was reflected in the rise in mean heart rate, LF power (nu %), LF/HF ratio, LF and LF% during *Bhastrika* practice, indicating the activation of the sympathetic nervous system. The LF/HF ratio increase was highly statistically significant during the manoeuver, while HF and HF% decreased significantly (p<0.001), showing parasympathetic withdrawal. Literature suggests that fast-paced *Bhastrika* (>60 breaths/minute) increases heart rate, rate pressure product and double product, indicating an increased load on the heart and a subsequent reduction in HRV [20]. In contrast, slow-paced *Bhastrika* (6 breaths/minute) leads to reductions in blood pressure and heart rate, indicative of parasympathetic activation [21]. Therefore, the cardiovascular impact of rapid *Bhastrika* observed in our study may be linked to the increased burden on the heart and decreased HRV due to heightened sympathetic activity and reduced parasympathetic response. The changes in HRV during rapid *Bhastrika* are similar to those observed during physical exercise, making it a potential alternative for individuals who are unwilling or unable to engage in traditional exercise [22].

In our study, the increase in heart rate observed during *Bhastrikriya* was sustained and did not return to normal resting levels even after five minutes. This may be attributed to the increased breath rate during *Bhastrika*, sympathetic stimulation and parasympathetic withdrawal. These values remained higher than baseline even five minutes post-practice. *Bhastrika Pranayama*, characterized by vigorous inhalation and exhalation, has also been associated with increases in blood pressure and heart rate, decreased CO2 levels, vasoconstriction and reduced cerebral blood flow (CBF) in intracranial arteries. Supporting this theory, research has shown that different *pranayamas* have opposing effects on cerebral hemodynamics when examined using continuous transcranial Doppler (TCD) [23]. Specifically, *Bhastrika Pranayama* has been found to lower CBF while raising heart rate and blood pressure, leading to hypocapnia. Comparing *Bhastrika Pranayama* with Kapalbhati (KB), which involves single active inhalations followed by strong expirations, KB has been shown to increase mental activity. Both *Bhastrika* and KB share similarities in cerebral hemo-dynamics and EEG signals, as both can increase sympathetic activity.

## EEG and *Bhastrika*:

## Spectrum of brain activity:

During *Bhastrika*, the delta wave increased and continued to raise post-*pranayama*, indicating a sustained effect of the practice. In contrast, beta, theta, alpha and gamma waves decreased significantly during *Bhastrika* (Table 2). A similar study on Satyananda Yogis found that after four years of practice, theta and alpha (low-frequency oscillations) increased in the right superior frontal, right inferior frontal and right anterior temporal lobes. After 30 years, beta and gamma (high-frequency oscillations) increased in the same regions [24]. Our previous research has focused on yoga practitioners who have engaged in slow, deep breathing exercises and meditation for 10 to 16 years. These practices have been shown to enhance beta and gamma activity, with gamma oscillations linked to the brain's frontal default mode network, reflecting improved coherence observed in long-term mindfulness meditators [25, 26]. Other studies have shown that regular meditation practice leads to high-amplitude gamma synchronization across the frontal and parieto-temporal lobes in EEG recordings [27]. However, in our study, beta, theta, alpha and gamma waves decreased during *Bhastrika*. This may be due to the short duration of the *pranayama* in this acute study. Further research is needed to understand the long term effects of *Bhastrika* on the brain's EEG wave spectrum.

This study has some limitations, including (i) a limited sample size and (ii) the need for additional tests, such as baroreflex sensitivity. Therefore, further research with a larger sample size and objective criteria is needed for a clearer understanding. (iii) As this was an acute study, the effects of chronic *Bhastrika* practice need to be determined. Understanding the long-term impacts of *Bhastrika* on the brain's EEG wave spectrum and HRV requires more research. The HRV variations observed during fast-paced *Bhastrika* are comparable to those seen during physical exercise. *Bhastrika Pranayama* may offer benefits similar to those of physical exercise for individuals who are unwilling or unable to exercise. Regular practice of *Bhastrika Pranayama* can help manage anxiety by calming the body and mind, potentially improving heart and lung function, blood flow and cardiovascular health. *Bhastrika* strengthens the respiratory system, increases oxygen intake and detoxifies the lungs, making it beneficial for respiratory ailments like asthma and bronchitis.

## Conclusion:

The study demonstrates that *Bhastrika Pranayama* significantly affects autonomic nervous system function by increasing sympathetic activity and decreasing parasympathetic influence, as shown by changes in heart rate variability. Additionally, it alters EEG brain wave patterns, indicating shifts in cognitive processing and states of consciousness. These findings suggest that *Bhastrika Pranayama* may offer therapeutic benefits for stress management and mental clarity, but further research is needed to assess its long-term effects.

## Conflict of interest statement:

The authors declare that there are no conflicts of interest regarding the publication of this paper.

## Ethical statement:

This study was conducted in accordance with the ethical standards of the institute. Before the start of the research, the Institutional Human Ethics Committee provided its ethical permission through a letter with IHEC No. IM079 as the reference. Informed consent was obtained from all individual participants included in the study.

## Figures and Tables

**Table 1:** Heart Rate Variability parameters before, during and after *Bhastrikriya Pranayama*

**Parameter**	**Pre *Bhastrika***	**During *Bhastrika***	**Post *Bhastrika***	**Repeated ANNOVA**	**Post Hoc Tests Green House Gelsser/ Huynh Feldt**		
					**A-B Pre/during**	**B-C During/post**	**A-C Pre/post**
HR	72.6	72.85	75.8	F (2, 38) 0.001*	1	0.002*	0.006*
SDNN	39.15	45.19	45.63	F (2, 38) 0.011*	0.028*	0.024*	1
pNN50	17.1	20.36	9.65	F (2, 38) <.001*	0.562	0.012*	<0.001*
RMSSD	35.43	37.73	29.98	F (2, 38) <0.001*	0.278	<.001*	<.001*
Total Power	1482.25	1815.6	1752.65	F (2, 38) 0.179	0.247	1	0.467
HF	524.2	372.7	367.85	F (2,38) <.001*	0.001*	1	<.001*
LF	422.2	615.65	430.7	F (2,38) 0.141	0.241	1.717	1
VLF Frequency	508.35	827.35	982.25	F (1.45, 27.555) .001**; €=0.725	0.014*	0.458	<.001*
LF%	27.73	28.26	26.68	F (2,38) 0.934	1	1	1
HF%	39.263	23.368	18.31	F (2, 36) < 0.001***; €=0.797	<.001*	0.217	<.001*
VLF%	32.68	48.42	55.05	F (2, 26) < .001***	<.001*	0.275	<.001*
LF/HF Ratio	0.914	1.817	1.335	F (2,38) 0.012*	0.010*	0.304	0.454
*Significance p<0.05; ** Test of sphericity; Greenhouse-Geisser test € *** Huynh-Feldt test €
HR-Heart Rate; SDNN-Standard deviation of the NN (R-R) intervals; pNN50-The proportion of NN50 divided by the total number of NN
(R-R) intervals; RMSSD: Root mean square of the successive differences; HF- High Frequency; LF- Low Frequency; VLF: Very Low Frequency.

**Table 2:** Comparison of EEG parameters before, during and after *Bhastrika*

**Parameter**	**Pre *Bhastrika***	**During *Bhastrika***	**Post *Bhastrika***	**Repeated ANNOVA**	**Post Hoc Tests Green House Gelsser/ Huynh Feldt**
					**A-B Pre/during**	**B-C During/post**	**A-C Pre/post**
Theta waves	20.47	14.89	13.84	F (2, 36) 0.002*	0.016*	1	0.003*
Alpha waves	15.78	13.84	6.78	F (1.36, 24.502) 0.002** €=0.681**	1	0.005*	<.001
Beta waves	12	8.47	5.57	F (1.305, 23.49) 0.020** €=0.653**	0.232	0.432	0.006*
Gamma waves	6.684	4.579	4	F (2, 36) <.001*	0.007	1	<.001*
Delta waves	45.105	58.474	69.895	F (1.535, 27.625) <.001 ** €=0.767**	0.014*	0.042*	<.001*
*Significance p<0.05; ** Test of sphericity; Greenhouse-Geisser test €
